# Colored Anodic Titania Thin Layers Involving Various Deep Eutectic Solvent Formulations—Evaluation of Corrosion Behavior

**DOI:** 10.3390/ma19061087

**Published:** 2026-03-12

**Authors:** Sabrina State (Rosoiu), Adrian-Cristian Manea, Oana Brincoveanu, Veronica Anastasoaie, Liana Anicai

**Affiliations:** 1 Faculty of Medical Engineering, National University of Science and Technology Politehnica Bucharest, 132 Calea Grivitei, 010737 Bucharest, Romania; sabrina.rosoiu@upb.ro; 2 National Institute of Research and Development in Microtechnologies-IMT, 126A Erou Iancu Nicolae Str., 077190 Voluntari-Ilfov, Romania; oana.brincoveanu@imt.ro (O.B.); veronica.anastasoaie@imt.ro (V.A.); 3 Department of Inorganic Chemistry, Physical Chemistryand Electrochemistry, National University of Science and Technology Politehnica Bucharest, 132 Calea Grivitei, 010737 Bucharest, Romania; adrianmanea@catedra.chfiz.pub.ro; 4 Center of Surface Science and Nanotechnology, National University of Science and Technology Politehnica Bucharest, Splaiul Independentei 313, 060042 Bucharest, Romania

**Keywords:** titanium, anodic oxidation, deep eutectic solvents, colored films, corrosion performance

## Abstract

**Highlights:**

**What are the main findings?**

- Anodic oxidation of Ti at constant applied voltage using various deep eutectic solvent formulations successfully produced a wide color palette.
- Growth rates between 1.6 and 2.1 nm/V have been determined, with good linear regression coefficients.
- Anodic layers synthesized in choline–chloride–oxalic acid and choline dihydrogen citrate–oxalic acid–ethylene glycol eutectic mixtures at 30 V for 3 min showed the best corrosion performance during long-term exposure in 0.5 M NaCl for 240 h and Hank’s biological solution for 96 h.

**What are the implications of the main findings?**

- Deep eutectic solvents may represent an attractive, environmentally friendly route to anodically color Ti substrates.
- The selected DES-based electrolytes can be applied for short durations (i.e., 3 min) at room temperature.
- The colored anodic titanium oxide thin layers could provide adequate corrosion protection during long-term exposure in various aggressive environments.

**Abstract:**

This paper reports initial experimental results related to the preparation of colored anodic titania thin layers using various deep eutectic solvent (DES)-based formulations. Electrolytes based on choline dihydrogen citrate–oxalic acid–ethylene glycol (1:1:1 molar ratio), choline chloride–oxalic acid (1:1 molar ratio) and choline chloride–lactic acid (1:2 molar ratio) eutectic mixtures were investigated. The anodization has been performed at constant voltage in a range of 10–100 V for various periods of time between 1 and 5 min at room temperature under mild stirring. A brief description of anodization procedures, as well as of some characteristics, from appearance and morphological viewpoints, is presented. A quantitative analysis of color characteristics in relation to the DES-based electrolyte and applied voltage using the CIELAB system is also discussed. The achieved chromatic scale follows this order of colors: golden—blue—light blue—light blue/green—pink—violet. This depends on the applied potential and the DES-based electrolyte. The films present a relatively high brightness and color saturation. The hue vs. anodization voltage diagrams suggest an almost linear dependence of the oxide growth measured against the applied voltage. The corrosion performance has been assessed through continuous immersion tests in (i) 0.5 M NaCl for 240 h and (ii) Hank’s biological solution for 96 h with intermediate visual examinations and recording corrosion potential, as well as potentiodynamic polarization curves and impedance spectra at open circuit potential. Different corrosion performances are discussed considering the aggressive medium involved and the used DES-based systems.

## 1. Introduction

Owing to their low density, biocompatibility characteristics, high strength-to-weight ratio, and ability to withstand higher temperatures (up to 900 °C) and to provide significant corrosion resistance, titanium (Ti) and its alloys represent metallic substrates with wide applications in the aerospace, automotive, military, and medical fields, as well as in the production of high-performance consumer goods [1,2,3].

Effective application of Ti often requires the use of appropriate surface modification methods. The anodic oxidation of Ti, as one of the most applied procedures, results in the thickening of the naturally developed thin transparent oxide layer, up to a desired value depending on the final application. This process is relatively easy to use and does not require high-tech instrumentation. Moreover, as the light interacts with the surface oxide layers, a large palette of interference colors on Ti surfaces can be produced, with attractive aesthetic properties that are of interest among architects, designers and jewelry designers [4,5,6,7]. Besides their aesthetic characteristics, the colored anodic titania layers are used to provide superior anticorrosive protection, mechanical and wear resistance, and improved biocompatibility. Moreover, for medical applications, such as dental and orthopedic implants, the colors allow for the identification of components (i.e., various sizes of medical screws; various instruments and apparatus components) without affecting the properties of the metallic substrate [2,7,8]. The brightness of the layers improves their decorative characteristics, contributes to a better corrosion resistance, and allows for an enhanced visibility for identification in medical and industrial applications [2,8,9].

A wide range of electrolytes has been used for the anodic coloring of Ti metallic substrates, primarily composed of aqueous acidic solutions based on sulfuric, phosphoric, or hydrofluoric acid and occasionally their salts [5,6,10,11]. As they involve aggressive inorganic acids that pose safety risks, researchers have focused on the development of “greener” electrolytes. Diamanti et al. [12] selected two electrolytes with no toxicity concerns based on (NH_4_)_2_SO_4_, a common fertilizer, and on NH_4_BF_4_, which were found to be suitable for architectural applications of anodized titanium and for semi-noble jewelry production, respectively. Zaniolo et al. [13] reported Ti anodization in electrolytes based on acetic acid and sodium bicarbonate as green alternatives. A wide range of colors was produced, influenced by the applied potential and by the electrolyte characteristics and concentration. Michalska-Domańska et al. [14] investigated the growth of colored nanotubular anodic titania using an ethanol-based electrolyte, with deionized water content ranging from 2 to 10 vol.% and a constant concentration of NH_4_F of 0.3 M at anodization voltages from 30 V to 60 V. The authors reported that anodic titanium oxide formed in an electrolyte containing 2 vol.% of water consistently exhibited a blue color, regardless of the applied voltage. In contrast, samples produced from other electrolyte compositions showed voltage-dependent color variations. They suggested that the resulting structural color arises from the combined influence of the nanotube diameter and the oxide layer thickness [14].

Considerable attention is now directed toward replacing aggressive and toxic chemical compounds with less harmful ones. In this context, the use of the so-called “deep eutectic solvents” (DESs) or “ionic liquid analogues” (ILAs) has increased. These media are typically defined as eutectic mixtures of quaternary ammonium salts (e.g., choline chloride and choline dihydrogen citrate) with hydrogen bond donor species such as amides, carboxylic acids or alcohols. They are potentially recyclable and biodegradable, with no harm to human health. In addition, DESs can be easily synthesized, show no reactivity in the presence of air humidity, and offer cost and environmental advantages [15,16,17].

Most studies report the use of DESs as electrolytic media for metal and alloy electrodeposition and for metal oxide preparation [18,19,20,21].

Few papers have addressed the anodization processes of valve metals in DESs. Fernandes et al. [22] recently reported the preparation of porous anodic oxide films on Al in ionic liquids consisting of eutectic mixtures of choline dihydrogen citrate, both with oxalic acid and with isopropyl alcohol and ethylene glycol. The procedure allowed for the synthesis of chloride-free ionic-liquid-based electrolytes, adequate for processing reactive metals such as Al. Uniform, homogeneous, and golden anodic alumina layers were prepared both in potentiostatic and galvanostatic conditions, at relatively high temperatures between 45 °C and 80 °C. Chen et al. [23] showed the formation of nanobamboos, i.e., nanotubes decorated with periodic exterior rings through the electrochemical spontaneous oscillation of current during Ti anodization, by adding 2–10% deep eutectic solvent (DES), consisting of a 1:1 molar ratio mixture of choline chloride–succinic acid, to a fluoride-containing aqueous electrolyte.

Winiarski et al. [24,25] studied Ti anodization in eutectic mixtures of choline dihydrogen citrate with oxalic acid at constant voltages between 10 V and 30 V at 60 °C for 10 min. The formed thin oxide films presented a porous morphology that exhibited various colors depending on the applied voltage value. In addition, the authors assessed the corrosion performance of the anodized layers in 0.05 M NaCl solution through linear polarization resistance (LPR) and electrochemical impedance spectroscopy (EIS) techniques. During the interpretation of the impedance spectra, the anodic layers were described using the two-layer model. It was assumed that the inner layer formed directly on the surface of metallic titanium was responsible for the barrier properties (resistance of 2.8 Mohm cm^2^). The porous outer layer formed on it had a much lower corrosion resistance, i.e., 800–1300 ohm cm^2^ [24]. Lu et al. [26] reported the formation of brightly saturated oxide films on Ti sheet surfaces via anodic oxidation under galvanostatic conditions (1 mA cm^−2^ at 30 °C for 15 min) in eutectic mixtures of choline chloride–ethylene glycol (1:2 molar ratio) in the presence of various water contents (from 0 wt.% to 100 wt.% in the DES) to enhance decorative properties and corrosion resistance. The authors found that the increase in water content facilitated a large range of developed colors with better reproducibility. They noticed a critical water content of 20 wt.%, which affected the formation and characteristics of the anodic layers. In addition, the EIS and potentiodynamic polarization tests performed in simulated artificial sweat solution (0.036 wt.% NH_3_ and 0.5 wt.% NaCl) showed a significant increase in impedance and a decrease in the corrosion current density of colored Ti sheets compared to untreated ones, suggesting an improvement in corrosion resistance.

Considering all presented above, this paper aims to investigate, for the first time, the use of novel DES-based formulations for the preparation of colored anodic titania thin layers. Eutectic mixtures of choline chloride–oxalic acid (1:1 molar ratio), choline chloride–lactic acid (1:2 molar ratio), and choline dihydrogen citrate–oxalic acid–ethylene glycol (1:1:1 molar ratio) are considered. The influence of an extended range of anodizing voltage (between 10 and 80 V) on the films’ morphology and color development is presented. To the best of our knowledge, the anodic coloring of Ti for short process durations (i.e., several minutes) at temperatures of 25–29 °C in DES-based electrolytes (as are currently applied in commercial processes) has not been clearly discussed.

In addition, the overall corrosion performance of the formed anodic titania layers during long-term exposure to various aggressive media, including 0.5 M NaCl aqueous solution and Hank’s biological solution, is reported. The differences in corrosion behavior are examined considering the involved aggressive medium and the used DES-based systems.

## 2. Materials and Methods

Table 1 summarizes the compositions of the ionic liquids synthesized from choline-based salts. All chemical reagents—choline chloride (HOC_2_H_4_N(CH_3_)_3_Cl) (ChCl) (Merck, Darmstadt, Germany, >98%), choline dihydrogen citrate (denoted as ChCit, ≥98%, Aldrich, Washington DC, USA), oxalic acid dihydrate (OxAc, ≥99%, Aldrich, Washington DC, USA), ethylene glycol (EG, 99.0%, Silal Trading, Bucharest, Romania) and lactic acid (LA, ≥80%, RemedLab, Bucharest, Romania)—were used as received.

Choline chloride–lactic acid (denoted ChCl-LA, 1:2 molar ratio) and choline chloride–oxalic acid (denoted ChCl-OxAc, 1:1 molar ratio) eutectic mixtures were obtained by gently stirring the components while heating them to 80–100 °C, continuing the process until a homogeneous, colorless liquid resulted. The mixture of choline citrate, oxalic acid and ethylene glycol (denoted ChCit-OxAc-EG, 1:1:1 molar ratio) was heated between 100 °C and 120 °C to facilitate the formation of a transparent liquid. An additional ethylene glycol volume was then added to that mixture to prepare the ChCit-OxAc-EG/EG electrolyte, as shown in Table 1.

The viscosity of the prepared DES-based electrolytes was determined using an Anton Paar (model AMVn) automatic microviscosimeter, equipped with an integrated Peltier thermostat with ±0.05 °C accuracy for the temperature control. In addition, Karl Fischer titration (TitroLine^®^ 7500 KF trace titrator, SI Analytics, Mainz, Germany) was used to determine their water content.

The anodic oxidation experiments were conducted under stationary, open-system conditions using a DC power supply (N5769A Power Supply, Agilent Technologies, Santa Clara, CA, USA, 0–15 A, 0–100 V). The electrochemical cell operated in a two-electrode set-up and contained ≈100 mL of ionic liquid as electrolyte.

Ti electrodes (commercial Ti foil #0.1 mm, min. 99.9% purity, with an exposed surface area of 10 cm^2^; Ti screws of 5 mm diameter, with a geometrical area of 10.4 cm^2^ (provided by Steiger Galvanotechnique SA, Châtel-St-Denis, Switzerland)) were used and measured against a Ti counter electrode. The anodization process was performed under potentiostatic conditions, and the voltage was varied between 10 and 90 V for time intervals between 1 and 5 min., at 27 ± 2 °C under mild stirring or in stationary conditions. Before the anodic treatment, the Ti surface underwent a 30 s degreasing step in acetone, followed by a surface treatment consisting of a chemical pickling using a mixture of HNO_3_–water (1:1 vol.) solution containing 2% HF for 30 s at room temperature, followed by water rinsing and drying.

Following the anodic oxidation step, the anodized samples were washed with ethanol and distilled water and subsequently dried before any additional analysis.

Cyclic voltammetry (CV) was employed to investigate the anodic response of the Ti electrode in the synthesized electrolytes, using a three-electrode glass cell comprising a 1 cm^2^ Ti working electrode, a platinum counter electrode, and a silver wire as quasi-reference electrode. The CV studies were performed using a PARSTAT 4000 potentiostat, Ametek, Berwyn, PA, USA controlled with the VersaStudio software, 2.1 version. A Nova NanoSEM 630 (FEI, Hillsboro, OR, USA), operated at 10 kV and coupled with an X-ray spectrometer (EDX, Smart Insight AMETEK, Mahwah, NJ, USA), was used to analyze the morphology of the Ti surfaces. The topography and roughness properties were investigated via a Scanning Near-Field Optical Microscope—Witec alpha 300S (Ulm, Germany)/with Raman module for AFM/Raman/imaging (Witec, Ulm, Germany). The AFM measurements were performed in tapping mode using a commercially available cone-shaped tip from monocrystalline silicon mounted on a cantilever with a stiffness of about 4.2 N/m. All images (512 × 512 lines) were acquired in ambient conditions with 4 s per line. The root mean square (RMS) and the average (Ra) roughness parameters were calculated from the acquired topographic images using the processing software.

The quantitative analysis of color characteristics was performed on anodized specimens. Color was measured using a UV–Vis spectrophotometer (ThermoScientific^TM^ Evolution 220, Madison, WI, USA light source: D65), and the values were processed by applying the CIELAB system (VisionLiteColorCalc Software, firmware 3.0.0.109), in which each color is represented by a point in the color space, which, in turn, is identified by three coordinates, L*, a* and b*, as defined by the International Commission on Illumination (Commission international de l’éclairage) in 1976. L* represents lightness, with 0 corresponding to black or darkness, and 100 corresponding to white or lightness. The values a* and b* are the chromatic coordinates, respectively varying between green (−a*) and red (+a*) and between blue (−b*) and yellow (+b*), and determine the actual color of the examined material. The hue angle, defined as the angular coordinate obtained by converting the rectangular coordinates a* and b* in cylindrical coordinates (tan h = b*/a*), is also an important factor used to measure the color difference from one specimen to another.

Besides color attributes, from spectrophotometric analyses, it was also possible to determine the thickness of the anodic oxides by identifying the position of the reflectance curve maxima and minima [5,6,27] and using the refractive index of the titanium oxide film, which varies with wavelength, according to the equation below [7,24,28]:

(1) $$n_{0}^{2}=5.913+\frac{2.441\times 10^{7}}{\lambda_{max}^{2}-0.803\times 10^{7}}$$

where λ_max_ is the wavelength maxima in the reflection spectra, and *n*_0_ is the refractive index of the film.

The corrosion performance of the colored anodized titania thin films was investigated using accelerated laboratory tests consisting of: (i) continuous immersion tests in 0.5 M NaCl for 240 h and Hank’s biological solution (composition shown in Table 2) [29] for 96 h with periodical visual observations accompanied by corrosion-potential measurements; (ii) recording the potentiodynamic polarization curves at a scan rate of 1 mV s^−1^; and (iii) the impedance spectra (EIS) at open circuit potential, measured against an Ag/AgCl reference electrode and using a Pt counter electrode. For both electrochemical investigations, the working electrode, WE, was represented by the colored anodized titania thin films prepared at 30 V in the abovementioned DES-based electrolytes (see Table 1) for 3 min., with a geometrical constant surface of 0.785 cm^2^. A minimum of 3 anodized pieces (40 × 30 mm) for each DES-based system were subjected to the tests. EIS spectra were recorded with 10 mV a.c. voltage within a 100 kHz–100 mHz frequency range, and the ZView 2.4 software from Scribner Association Inc., Derek Johnson, was used to process the experimental data. An Autolab PGSTAT 12 potentiostat controlled with the GPES software, 4.9.007 version was used during this experimental sequence.

## 3. Results and Discussion

The prepared ionic liquid-based electrolytes showed a transparent and colorless appearance. Based on Karl Fischer titration, the water contents of the ChCl-LA, ChCl-OxAc and ChCit-OxAc-EG/EG systems were found to be around 94,000 ppm (≈9.4%), 57,000 ppm (≈5.7%) and 87,000 ppm (≈8.7%), respectively. In addition, viscosity values of about 120 mPa s, 125 mPa s and 210 mPa s at a temperature of 30 ± 2 °C were determined for ChCl-LA, ChCl-OxAc and ChCit-OxAc-EG/EG, respectively.

### 3.1. Electrochemical Studies

**Figure 1:**
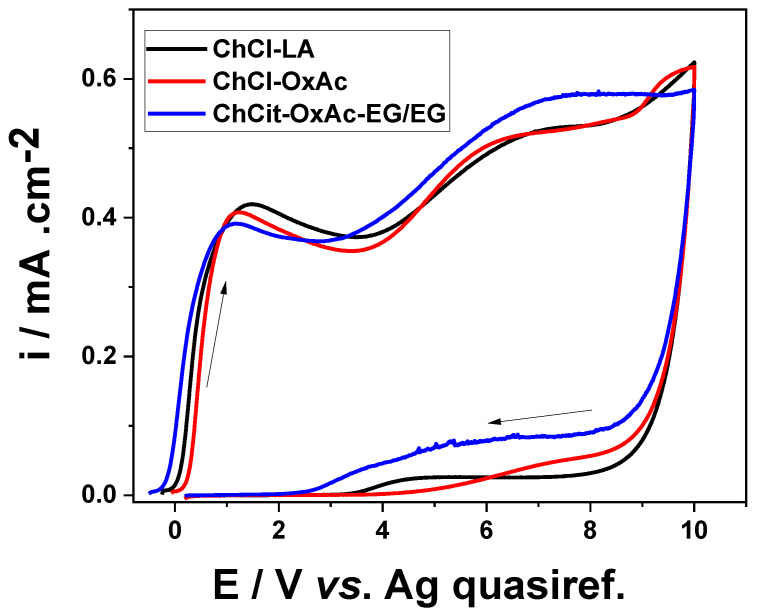
Cyclic voltammograms of Ti working electrode (1 cm^2^) in different DES-based electrolytes (25 °C; scanning rate: 100 mV s^−1^).

Figure 1 displays the cyclic voltammograms recorded for the three ionic liquid-based electrolytes, using a scan rate of 100 mV s^−1^. It can be seen that the CV shape is, in general, similar to the physical model of the high-field conduction behavior of the oxide growth, which is a characteristic kinetic behavior of valve metals [30,31]. A significant rise in current density can be seen as the potential moves toward more anodic values, regardless of the involved electrolyte type.

Three distinct potential regions can be identified: (i) a rapid increase in current is observed up to an anodic potential in the range 1–1.4 V, which may be ascribed to the development of the barrier layer, and (ii) a small plateau centered at about 2.8 V, 3.2 V and 3.3 V for the ChCit-OxAc-EG/EG, ChCl-OxAc and ChCl-LA electrolytes, respectively, usually assigned to the development the pores, followed by (iii) a progressive current increase as the anodic oxide continues to develop. During the backward sweep, the current density decreased markedly due to the blocking effect of the insulating anodic oxide layer. In addition, a small leakage current was noticed, suggesting the formation of a porous anodic oxide, allowing for, however, a certain electrolyte penetration at the metal/oxide interfaces.

According to the literature [32,33], a classical anodization scheme may be considered, consisting of ion formation (Equation (2)), followed by a reaction with O^2−^ caused by the deprotonation of H_2_O or OH^−^ from ethylene glycol (EG) under high field conditions (Equations (3)–(5)). Therefore, under anodic polarization and in the presence of water traces within the electrolyte, the following reactions may occur:

Ti ⟶ Ti^4+^ + 4e^−^ (2)

Ti + 2H_2_O ⟶ TiO_2_ +4H^+^ + 4e^−^ (3)

Ti^4+^ + 4H_2_O ⟶ Ti(OH)_4_ + 4H^+^ (4)

Ti(OH)_4_ ⟶ TiO_2_ + 2H_2_O (5)

Simultaneously, at the cathode, the counter reaction occurs, which is hydrogen evolution:

4H_2_O +4e^−^ ⟶ 2H_2_ + 4OH^−^ (6)

### 3.2. Morphological Characterization of the Colored Anodic Titanium Oxide Thin Films

The Ti substrates were anodically oxidized in the DES systems described in Table 1 at room temperature, with processing times between 1 and 5 min. For shorter periods of 1–2 min, colorless films were obtained.

As the duration increased to 3 min., the layers showed well-defined colors, whose evolution depended on the applied voltage, as expected. The anodization period increases to 5 min did not evidence any change in the film characteristics or the obtained color. Therefore, an anodization period of 3 min was selected to be applied during the experiments.

Uniform and adherent layers were obtained under the investigated operation conditions.

**Figure 2:**
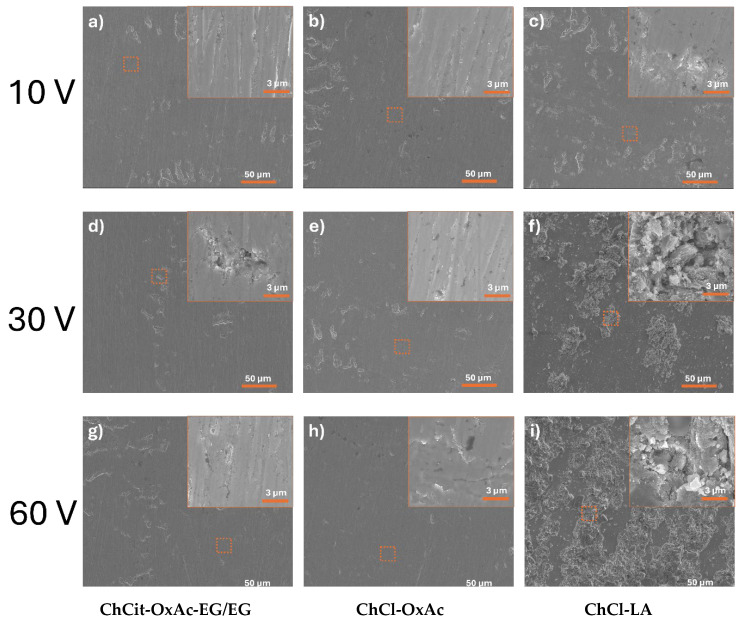
SEM micrographs at different magnifications (×1000 and ×25,000) of the colored films on Ti during anodization involving ChCit-OxAc-EG/EG (**a**,**d**,**g**), ChCl-OxAc (**b**,**e**,**h**) and ChCl-LA (**c**,**f**,**i**) at 10 V (**a**–**c**), 30 V (**d**–**f**) and 60 V (**g**–**i**) (3 min anodization duration).

Examples of the recorded SEM micrographs of the colored Ti sheets anodized at various applied potentials for 3 min at room temperature in the investigated DES-based electrolytes are presented in Figure 2.

For the oxalic-acid-based electrolytes, namely ChCit-OxAc-EG/EG (Figure 2a,d,g) and ChCl-OxAc (Figure 2b,e,h), the anodized surfaces exhibit relatively smooth and compact morphologies, relatively independent of the applied anodization potential.

In both cases, the surfaces display noticeable etching marks and sporadically distributed small pits, more pronounced for higher anodization voltages of 30 V and 60 V. The change in the hydrogen bond acceptor (HBA) from choline citrate (in ChCit-OxAc-EG/EG) to choline chloride (in ChCl-OxAc) does not result in significant morphological differences, suggesting similar anodization behavior in oxalic-acid-containing DESs under the investigated conditions.

In contrast, anodization in the lactic-acid-based electrolytes (ChCl-LA; Figure 2c,f,i) leads to markedly different surface morphologies, characterized by strong corrugated and highly irregular surfaces. In this system, the applied potential has a pronounced effect on the surface evolution. At 10 V, the surface is dominated by irregular pits and depressions. Increasing the potential to 30 V promotes the formation of elongated nanostructured features, while at 60 V, the surface develops more complex architectures, including partially developed tubular or pore-like nanostructures. Liu et al. [34] assigned the features of the anodic oxides to the bubbles generated from oxygen evolution, which become more intense as the anodization voltage increases.

**Figure 3:**
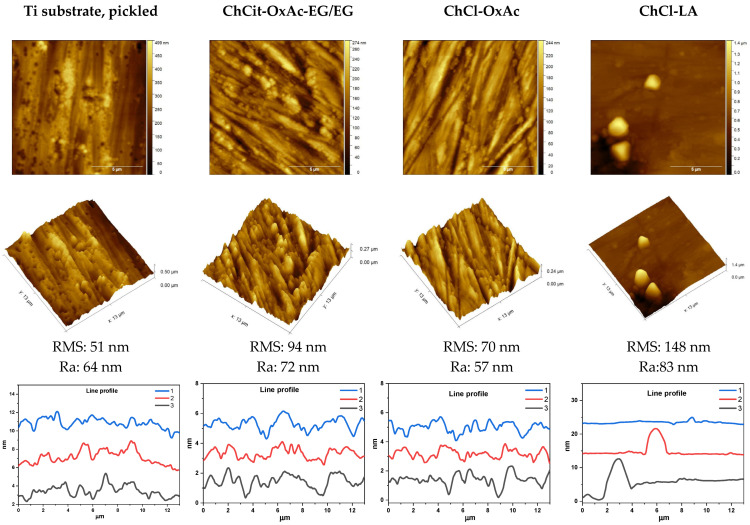
AFM images of etched Ti substrate and of the colored films on Ti during anodization in the investigated electrolytes at a constant voltage of 30 V for 3 min.

AFM images of the pickled Ti substrate (before anodization process) and of the colored layers in the investigated electrolytes at an anodization voltage of 30 V for 3 min are illustrated in Figure 3. Data regarding average roughness (Ra) and root mean square roughness (RMS) were calculated based on the entire test area. The surface morphology of the Ti foils after the pickling step shows significant heterogeneity, consisting mainly of directionally aligned scratches and microporous structures with an average roughness of 64 nm.

After anodizing at 30 V in ChCit-OxAc-EG/EG electrolyte, a uniform deposition layer with a granular structure aligned with the underlying metallic texture was observed, with an average roughness of 72 nm, higher than that of the sample prior to anodization. For the ChCl-OxAc DES-based electrolyte, the surface morphology of the titanium foil appears smoother and more compact, with an average roughness of 57 nm. The anodic layer is more homogeneous, characterized by undulating topography and visible granules, while retaining the fine scratches observed on the uncoated sample. A distinct morphological change was evident after anodization in the ChCl-LA electrolyte, where the average roughness reached 148 nm, reflecting the appearance of micron-scale deposits. The morphology shows the development of a substantially compact, heterogeneous deposit layer covering the scratches on the titanium foil, resulting in the formation of large particle aggregates that dominate the topography.

The three profiles in each graph are taken from different lateral positions, so their absolute vertical level (baseline) may differ. The profile analysis indicates that the roughness of the uncoated sample is determined mainly by the substrate base surface (pickling grooves), whereas the specimen obtained in the ChCit-OxAc-EG/EG system has a uniform texture at the nanometric scale, with reduced amplitude and roughness. The sample produced in the ChCl-OxAc system has a comparable texture, although slightly finer. In contrast, the sample obtained in the ChCl-LA electrolyte has uneven roughness, mainly characterized by aggregates.

The elemental composition of the colored titanium oxide layers obtained by anodic oxidation in the investigated DES-based electrolytes has been determined by EDX analysis and the results are presented in Table 3.

As illustrated in Table 3, the anodic films are mainly composed of titanium and oxygen, with low amounts of carbon. The traces of carbon suggest a partial incorporation of the DES-based electrolyte into the grown oxide layer.

### 3.3. Coloring Process Evaluation in Selected DES-Based Systems

**Figure 4:**
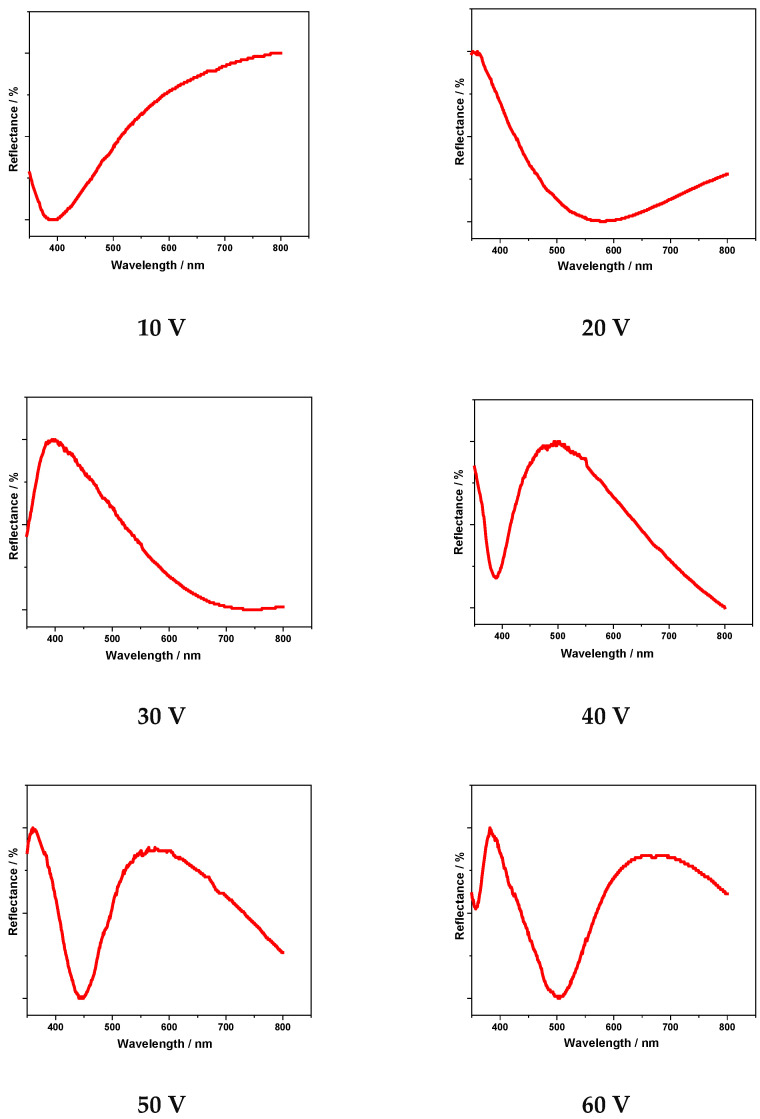
Reflectance spectra of interference colors obtained during Ti anodization in ChCl-OxAc electrolyte for different applied voltages.

The recorded reflectance spectra on pickled Ti substrates are illustrated in Figure 4 for the colored films anodized in ChCl-OxAc electrolyte by increasing the voltage from 10 V to 60 V for 3 min. In addition, the corresponding reflectance spectra related to the anodized specimens in the ChCl-LA and ChCit-OxAc-EG/EG electrolytes are presented in Figures S1 and S2 (Supplementary Materials).

The sinusoidal shape of the spectra, regardless of the used electrolyte, indicates the interference phenomena as the origin of the corresponding colors [5,10,26], in a similar manner noted during Ti anodic coloring in the aqueous electrolytes.

The overlap of light waves produces optical interference, in which some wavelengths are strengthened, and others are weakened, corresponding to constructive and destructive interference, respectively [5,25,35]. Equations (7) and (8), below, present the mathematical expressions describing constructive and destructive interference conditions:

– Constructive Interference:

2nd = m λ            m = 0, 1, 2, 3…(7)

– Destructive Interference:

2nd = (m + 1/2) λ    m = 0, 1, 2, 3…(8)

where n represents the refractive index of the titanium oxide, d is the thickness of the oxide layer, λ refers to the wavelength of light, and m is an integer. From the spectral curves and the calculated values of the refractive index according to Equation (1), the thickness of the anodic titanium oxide films can be calculated.

**Figure 5:**
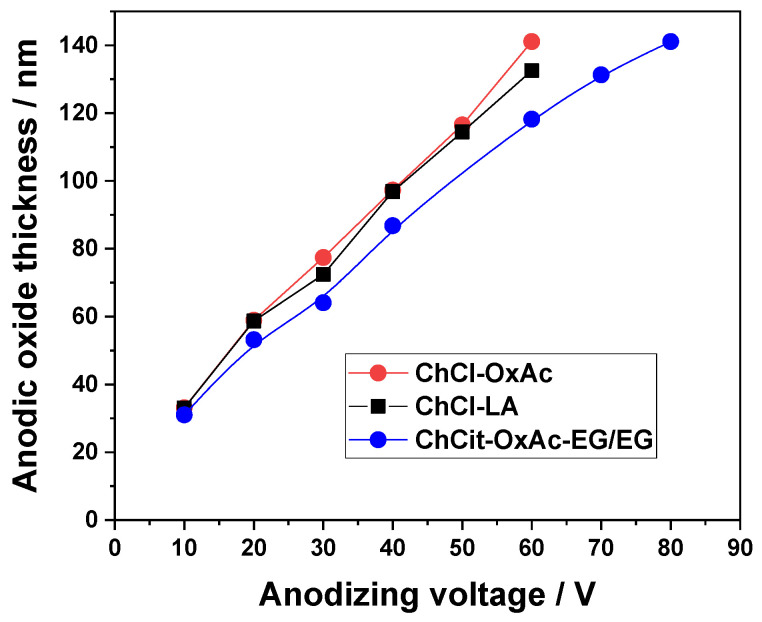
Thickness of oxide layers on Ti substrate vs. the applied anodizing voltage for various DES-based electrolytes.

In addition, the reflectance spectra exhibited an increasing number of maxima and minima, along with a displacement of these features toward longer wavelengths, indicating the thickening of the anodic oxide layer that generates the interference pattern. The calculated oxide thicknesses from reflectance spectra for Ti specimens after the anodization process in the investigated DES-based electrolytes are presented in Figure 5, for the applied voltage range.

As shown in Figure 5, a linear relationship between the oxide layer thickness and the applied anodization voltage was observed for all electrolytes examined. Growth rates between 1.6 and 2.1 nm/V have been determined, with good linear regression coefficients, as shown in Table 4. The relatively higher value of the oxide growth rate in ChCl-OxAc electrolyte could be related to its higher electrical conductivity (≈4.5 mS cm^−1^ at 25 °C) as compared to that of ChCl-LA (≈1.78–2 mS cm^−1^ at 25 °C) and ChCit-OxAc-EG/EG (≈1 mS cm^−1^ at 25 °C) [22,36,37].

A quantitative analysis of color characteristics against the involved DES-based electrolyte and the applied voltage has been performed by applying the CIELAB system. The achieved chromatic scale consisted of the following order of colors: gold—blue—light blue—light blue/green—pink—violet. This depended on the applied potential and the DES-based electrolyte. Examples of the color palette obtained by anodic oxidation in the investigated DES-based electrolytes on Ti foils and screws are illustrated in Figure S3.

**Figure 6:**
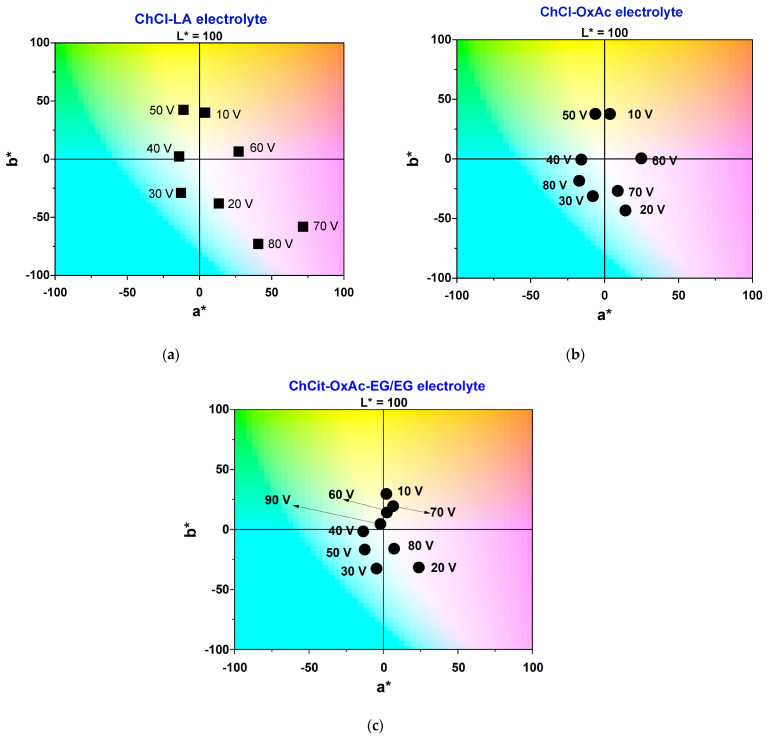
Chromaticity diagrams of anodic layers obtained in: (**a**) ChCl-LA, (**b**) ChCl-OxAc and (**c**) ChCit-OxAc-EG/EG electrolytes.

**Figure 7:**
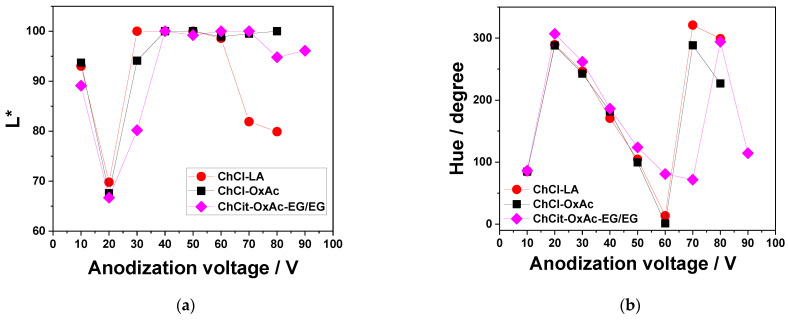
(**a**) The evolution of brightness (L*) and (**b**) of the hue angle, h, measured against the applied voltage during the Ti anodization process in the investigated DES-based electrolytes.

A further analysis of the color attribute variation compared with the applied voltage and electrolyte type is reported in Figure 6 and Figure 7. Spectrophotometric measurements of the specimens anodized in the ChCl-LA electrolyte showed bright surfaces, with L* values very close to 100 for anodization potentials of 10 V and between 30 and 50 V (see Figure 7a). In the a*-b* diagram (as shown in Figure 6a), the points representing sample colors are positioned relatively far from the inner shell, where the colors are less intense. A similar behavior was noticed when the anodization process occurred in the ChCl-OxAc system, as illustrated by the a*-b* diagram in Figure 6b. Bright surfaces with L* values higher than 90 were evidenced for anodization voltages of 10 V and higher than 30 V. Slightly more intense colors are found when the ChCit-OxAc-EG/EG electrolyte is used, as shown in Figure 6c, with a similar trend of brightness evolution.

Figure 7b illustrates the dependence of the hue angle against the applied anodization potential for the investigated electrolytes. In all cases, a periodic trend is shown, meaning that the same hue angles are repeatedly present as the applied potential increases. For example, at 10 V, the anodized surface is yellow, and the same color emerges once more when the voltage reaches 50 V for the ChCl-LA and ChCl-OxAc electrolytes and 60 V in the case of the ChCit-OxAc-EG/EG system. This periodicity results from the interference nature of the color formation and the linear relationship between anodization voltage and oxide-film thickness, which dictates the interference pattern [5,6,38,39,40].

### 3.4. Corrosion Behavior of Colored Anodic Titania Layers Obtained from DES-Based Electrolytes

To evaluate the corrosion resistance of the colored anodic titanium oxide layers produced in the various DES-based electrolytes, potentiodynamic polarization curves and electrochemical impedance spectra at open circuit potential have been recorded, involving two types of aggressive media, in free-aerated solutions, at room temperature. Therefore, the 0.5 M NaCl solution and Hank’s solution have been selected to perform experiments. All specimens subjected to the corrosion tests were prepared by applying an anodization potential of 30 V for 3 min at room temperature, involving ChCl-LA, Ch-Cl-OxAc and ChCit-OxAc-EG/EG electrolytes.

#### 3.4.1. Corrosion Behavior of Colored Anodic Titania Layers in 0.5 M NaCl

**Figure 8:**
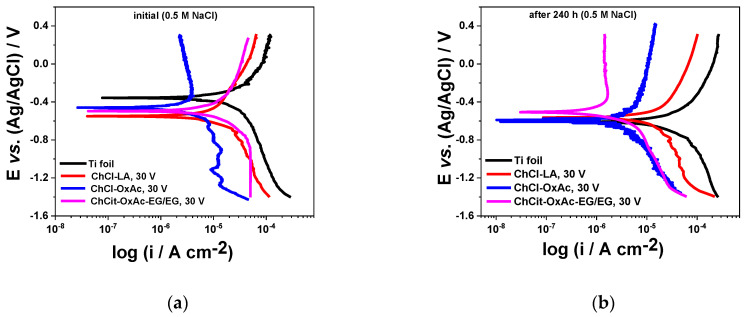
Polarization curves in semilogarithmic coordinates for anodic TiO_2_ thin layers obtained from DES-based systems during continuous immersion in 0.5 M NaCl: (**a**) initial and (**b**) after 240 h of conditioning (25 °C, 1 mV s^−1^).

Typical polarization curves in semilogarithmic form for the anodic TiO_2_ thin layers obtained in the ChCl-LA, Ch-Cl-OxAc and ChCit-OxAc-EG/EG electrolytes are presented in Figure 8. For reference, identical experiments were conducted on bare (unoxidized) Ti foil.

As shown in Figure 8a, immediately upon immersion, a displacement of the corrosion potential towards more negative values is noticed for all anodized layers, with about 100–200 mV, as compared to the non-anodized Ti foil. Both anodic and cathodic branches of the polarization curves for the anodized specimens show lower current densities, more pronounced for those produced in the Ch-Cl-OxAc system, due to their protective action. This decrease in the anodic current density for the anodized layers grown in Ch-Cl-OxAc could also be related to the formation of an additional passive film, which may reduce the corrosion process. This is a reasonable assumption in the case of passive layers on the Ti substrate [41,42].

After 240 h of conditioning, as presented in Figure 8b, the corrosion potential of the non-anodized Ti foil shifted to more negative values, also associated with an increase in the corrosion current density. The anodized specimens involving the ChCl-OxAc and ChCit-OxAc-EG/EG systems present more protective characteristics, reflected by lower values for the corrosion current density. In a similar manner, the anodic layers formed in ChCit-OxAc-EG/EG showed a significant decrease in the anodic current density as the applied potential was displaced towards more positive values, and this behavior could be attributed to the presence of a supplementary passive film, which may enhance the protective characteristics.

In the cathodic branches of the polarization curves in Figure 8, an initial linear current increase, up to around −1 ÷ −1.2 V, can be observed, likely attributable to an oxygen reduction reaction, whereas the further current rise at more cathodic potentials can be attributed to the hydrogen evolution reaction [43]. The relatively low values of the cathodic current density can also be seen. Under cathodic polarization, the composition of the anodic oxide layer can be changed as a result of hydrogen absorption according to the reaction TiO_2_ + H^+^ + e^−^ = TiOO(H)_ads_ [43,44].

The corrosion parameters (i_corr_ and E_corr_) were determined by applying Tafel extrapolation to the linear segments of both polarization branches, and the corresponding results are presented in Table 5.

The anodic oxide film provided the Ti substrate with increased protection against corrosion. Thus, immediately after immersion, values of ≈9 µA cm^−2^ for untreated Ti were determined, which decreased up to about 6 µA cm^−2^, 5.3 µA cm^−2^ and 2.1 µA cm^−2^ for the anodic layers formed in ChCit-OxAc-EG/EG, ChCl-LA and ChCl-OxAc, respectively. After 240 h of exposure, lower corrosion currents were noticed in the case of anodized films prepared in the ChCit-OxAc-EG/EG electrolyte. A lower performance in terms of i_corr_ showed the layers formed in the ChCl-LA and ChCl-OxAc systems. The different behavior of the specimens prepared in ChCl-OxAc and ChCl-LA is driven by their morphological differences: anodized layers in ChCl-OxAc likely exhibit localized structural integrity issues that act as pathways for the aggressive chloride anion to penetrate, while those obtained using ChCl-LA tend to exhibit a porous morphology that provides a greater total surface area or network of pores for ingress, rather than isolated defects. In the case of untreated Ti foil, an increase in i_corr_ is observed, associated with the progression of the metal dissolution process.

**Figure 9:**
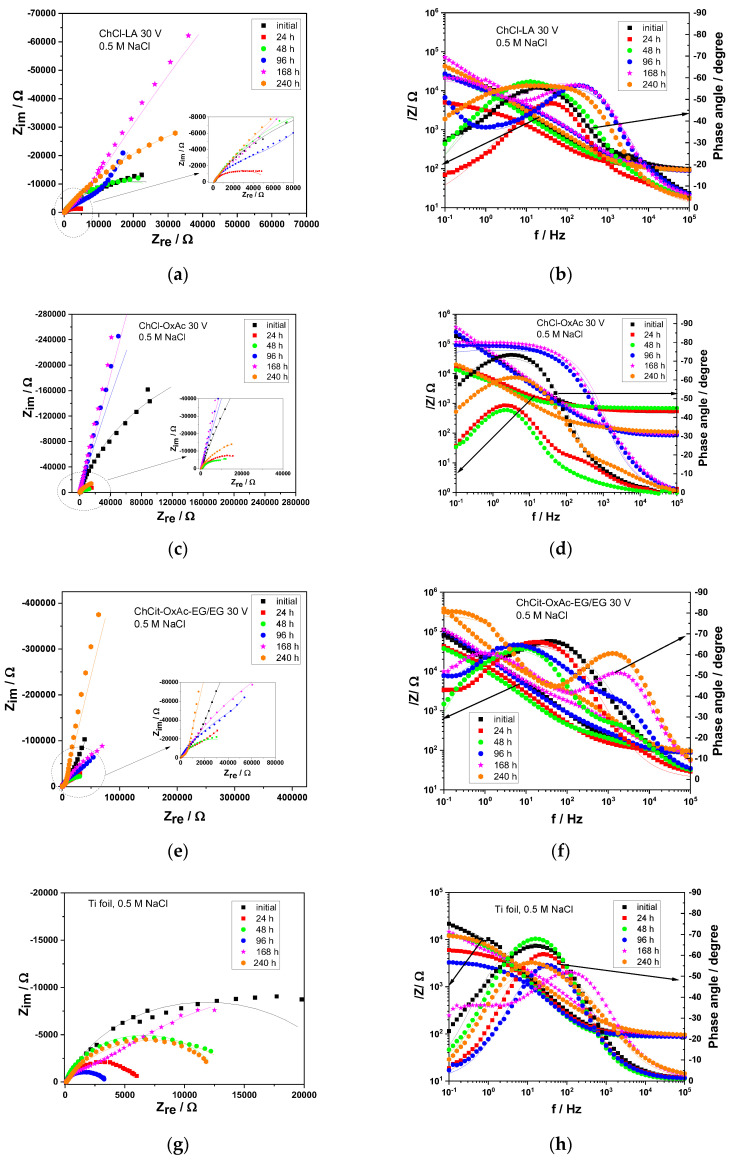
Comparative Nyquist and Bode plots in 0.5 M NaCl at open circuit potential, after various continuous immersion periods for anodic titanium oxide layers using (**a**,**b**) ChCl-LA, (**c**,**d**) ChCl-OxAc and (**e**,**f**) ChCit-OxAc-EG/EG systems, as well as for pure Ti foil (**g**,**h**). The solid lines represent the fits to the experimental data obtained using the equivalent circuits shown in Figure 10.

**Figure 10:**
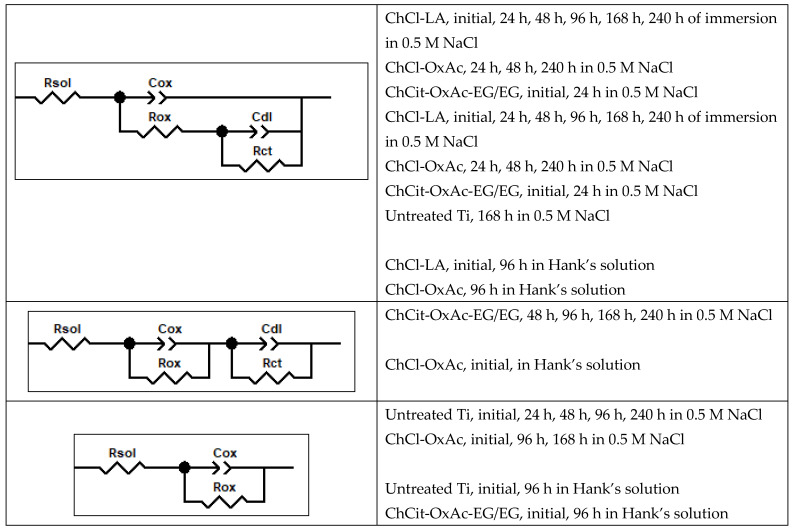
Electrical equivalent circuits used to fit the impedance spectra (Figures 9 and 13) recorded over 240 h of immersion in 0.5 M NaCl solution and 96 h in Hank’s solution. The significance of circuit components: Rsol—ohmic resistance of solution; Cdl—double-layer capacitance; Rct—charge transfer resistance; C(ox) and R(ox)—capacitance and ohmic resistance of the formed film.

The results shown in Figure 9 correspond to the electrochemical impedance spectra of the DES-derived colored anodic films and the untreated Ti substrate, recorded at open circuit potential (OCP) in the 0.5 M NaCl solution for different immersion durations. The proposed electrical equivalent circuits (EECs) used to describe their corrosion behavior are shown in Figure 10.

All Nyquist plots display a semicircle arc in the relatively high-frequency region. The diameter of the semicircles is generally associated with the resistance of the film and can be linked to corrosion behavior: a higher resistance corresponds to a reduced corrosion rate. Usually, the values of the phase angle at high frequency are also associated with higher impedance modulus, which could be an indicator of the protective properties of the layer.

In the case of the anodic layers prepared in the ChCl-LA electrolyte, two time constants are observed in the Bode plot (see Figure 9b) throughout the entire immersion period, which could be ascribed to the porous outer oxide layer and to the corrosion process at the oxide/metal interface. A reduction in both the phase angle and the impedance modulus is observed during the first 24 h of exposure, indicating possible deterioration of the layer. After that, an increase in the resistance occurs up to 168 h, and it could be ascribed to the additional formed passive film. Then, the impedance modulus decreased again up to 240 h of immersion, likely because the corrosion progressed as water and chloride ions penetrated the film.

For the anodized specimens using the ChCl-OxAc system, a capacitive behavior is suggested by phase angle values higher than −75° for the initial moment of immersion and between 96 and 168 h of exposure, followed by a decrease of up to −60° (see Figure 9d). This evolution suggests that a stable film formed on Ti in the used anodizing electrolyte; however, there are some defects present on the surface, which allowed for a certain amount of ingress from the aggressive solution. The impedance modulus decreased in the first 48 h of conditioning, followed by a continuous increase of up to 168 h of immersion, ascribed to a “healing” effect of the hydrated passive film [45]. Then, a slight decrease in the resistance occurred up to 240 h in a similar manner, as evidenced by the case of layers formed in the ChCl-LA system.

The specimens prepared in ChCit-OxAc-EG/EG represent two time constants in the Bode plot from Figure 9f. The time constant located in the high-frequency region could be related to the porous outer oxide layer, while the second one from the lower frequency could be attributed to a more compact inner layer. According to the literature [24,46,47], films grown on Ti substrates are composed of a porous outer layer and a more compact/barrier inner layer. The film resistance decreased after 48 h of immersion, and then, the increasing tendency continued up to the end of exposure, suggesting a good protective action.

The non-anodized Ti shows a successive change in the impedance modulus during the exposure period. The presence of the passive layer contributes to the increase in resistance, while its dissolution determines a corresponding decrease. Several equivalent circuits were considered to accurately reproduce the measured EIS data. The circuits offering the best fit, based on a lower chi-squared (i.e., χ^2^ in the range 4 × 10^−4^ ÷ 8 × 10^−3^) and minimum errors, were selected, as previously detailed in Figure 10. Constant phase elements (CPEs) were employed instead of capacitances [24,45,46,47] to more accurately represent the non-ideal capacitive behavior of the electrode/electrolyte interface. Rsol represents the ohmic resistance of the solution. C(ox) and R(ox) denote the capacitance and resistance of the formed film, while Cdl and Rct represent the double-layer capacitance and charge transfer resistance at the metal substrate.

**Figure 11:**
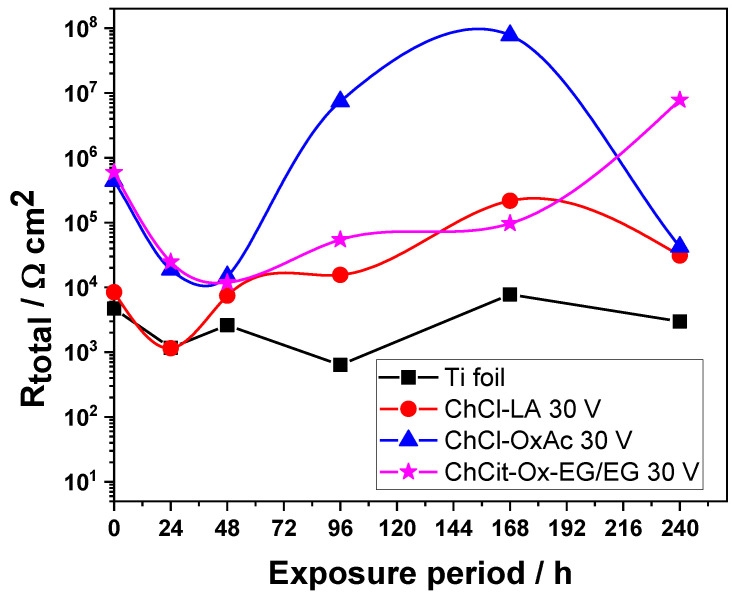
Evolution of Rtotal (R_total_ = R_ox_ + R_ct_) for anodic TiO_2_ thin layers obtained from DES-based electrolytes during continuous immersion test in 0.5 M NaCl.

The evolution of R(ox) and Rct measured against the immersion period for the anodic TiO_2_ thin layers obtained from the investigated DES systems is shown in Table 6 and Figure 11.

R_ox_ and R_ct_ show similar trends for all investigated anodic layers. However, the specimens anodized in the ChCl-OxAc and ChCit-OxAc-EG/EG electrolytes exhibited significantly higher values as compared to those synthesized in the ChCl-LA system.

Since various electrochemical parameters were employed to evaluate the processes occurring at the oxide layer/metal interface, the total resistance, R_total_, was calculated, and its evolution with immersion time is shown in Figure 11. R_total_ includes the resistance of the oxide layer and the charge transfer resistance, R_ct_; i.e., R_total_ = R_ox_ + R_ct_, and it could be considered a useful tool to evaluate the corrosion performance.

As shown in Figure 11, the best corrosion protection appears to be provided by the anodic layers formed in the ChCl-OxAc and ChCit-OxAc-EG/EG electrolytes. This behavior could be related to the layers’ morphology, as shown previously in Figure 2. The anodization process in oxalic-acid-based electrolytes, namely ChCl-OxAc and ChCit-OxAc-EG/EG, facilitated relatively smooth and compact morphologies that, in turn, could provide better corrosion protection. Conversely, the use of the ChCl-LA electrolyte produced a porous morphology, more susceptible to being penetrated by the aggressive chloride anion and, therefore, with fewer protective characteristics.

#### 3.4.2. Corrosion Behavior of Colored Anodic Titania Layers in Hank’s Solution

Hank’s solution reproduces the ionic environment of human blood plasma and thus serves as a standard medium in many biomedical experiments.

**Figure 12:**
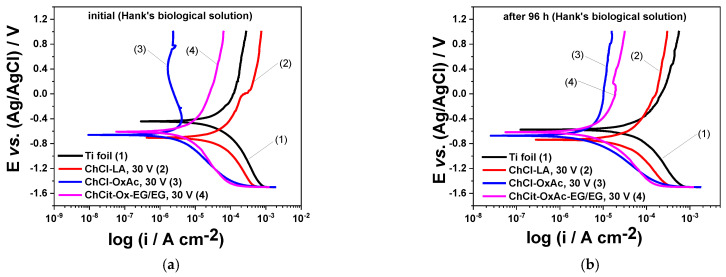
Polarization curves in semilogarithmic coordinates for anodic TiO_2_ thin layers obtained from DES-based electrolytes during continuous immersion in Hank’s biological solution: (**a**) initial and (**b**) after 96 h of conditioning (25 °C, 1 mV s^−1^).

Although Hank’s solution does not capture the full complexity of the physiological environment, it offers a sufficiently accurate ionic approximation for in vitro experiments [29,46]. Figure 12a,b comparatively present the recorded polarization plots in semilogarithmic coordinates corresponding to the anodic TiO_2_ thin layers obtained in the ChCl-LA, Ch-Cl-OxAc and ChCit-OxAc-EG/EG electrolytes, as well as to the untreated Ti foil for the initial moment and after 96 h of immersion in naturally aerated Hank’s solution.

As illustrated in Figure 12a, at the initial moment of immersion, all anodic oxide layers exhibit more electronegative corrosion potential values as compared to the non-anodized Ti foil. Both anodic and cathodic branches of the polarization curves for the anodized specimens using ChCl-OxAc and ChCit-OxAc-EG/EG systems show lower current densities as compared to those prepared in ChCl-LA electrolyte and to those of the untreated Ti foil. A significant decrease in the anodic current density as the applied potential was displaced towards more positive values was observed for anodic TiO_2_ layers obtained in ChCl-OxAc electrolytes, suggesting the formation of a supplementary protective film.

After 96 h of immersion, as presented in Figure 12b, the corrosion potential of all investigated specimens shifted to more negative values. Slightly lower corrosion current density values as compared to the initial moment of exposure were determined in the case of the non-anodized Ti foil and the specimens prepared in ChCl-LA electrolyte.

In the cathodic branches of the polarization curves in Figure 12, an initial linear current increase, up to around −1 ÷ −1.2 V, can be observed, likely attributable to an oxygen reduction reaction [43]. At more cathodic potentials, a fast rise in the current can be seen, likely attributable to a hydrogen evolution reaction [48].

The corrosion currents of the anodized specimens using the ChCl-OxAc and ChCit-OxAc-EG/EG systems remained almost the same. Their protective action is notable, mainly reflected by significantly lower currents on the anodic branches of the polarization curves.

Table 7 presents the corrosion current density, i_corr_, and corrosion potential, E_corr_, determined for each of the analyzed specimens.

The anodized layers on the Ti substrate improved its protective characteristics. Therefore, at the initial moment of immersion, values of ≈20 µA cm^−2^ for untreated Ti were determined, which decreased up to about 17 µA cm^−2^, 1 µA cm^−2^, and 2.4 µA cm^−2^ for the anodic layers formed in ChCl-LA, ChCl-OxAc, and ChCit-OxAc-EG/EG, respectively. After 96 h of conditioning, lower corrosion currents were noticed in the case of anodized films prepared in ChCl-LA, while slightly higher values were observed for those formed in ChCl-OxAc and ChCit-OxAc-EG/EG electrolytes, but they still provided the best corrosion performance. Such behavior might be attributed to defects within the layer that facilitated the penetration of the chloride-containing solution.

**Figure 13:**
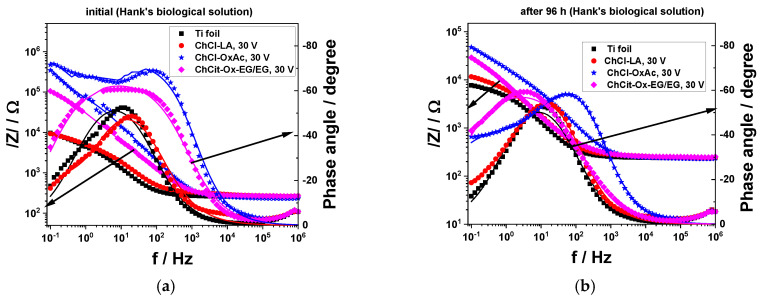
Comparative Bode plots in Hank’s biological solution at open circuit potential for anodic TiO_2_ thin layers obtained from DES-based electrolytes during continuous immersion in Hank’s biological solution: (**a**) initial and (**b**) after 96 h of conditioning. Solid lines are the fit to the measured points using the proposed equivalent circuits in Figure 10.

Figure 13 presents the electrochemical impedance spectra of the colored anodic titanium oxide layers using the investigated DES-based systems, as well as of the non-anodized Ti, recorded at the open circuit potential (OCP) in Hank’s solution for the initial and final moments of conditioning. The proposed electrical equivalent circuits used to describe their corrosion behavior are shown in Figure 10.

The EIS spectra evolution is consistent with the results provided by the polarization curves. Therefore, the anodic layers formed in ChCl-OxAc and ChCit-OxAc-EG/EG electrolytes provided better performance and present the highest values for the impedance modulus and the phase angle, both at the initial moment of immersion and after 96 h of exposure.

**Figure 14:**
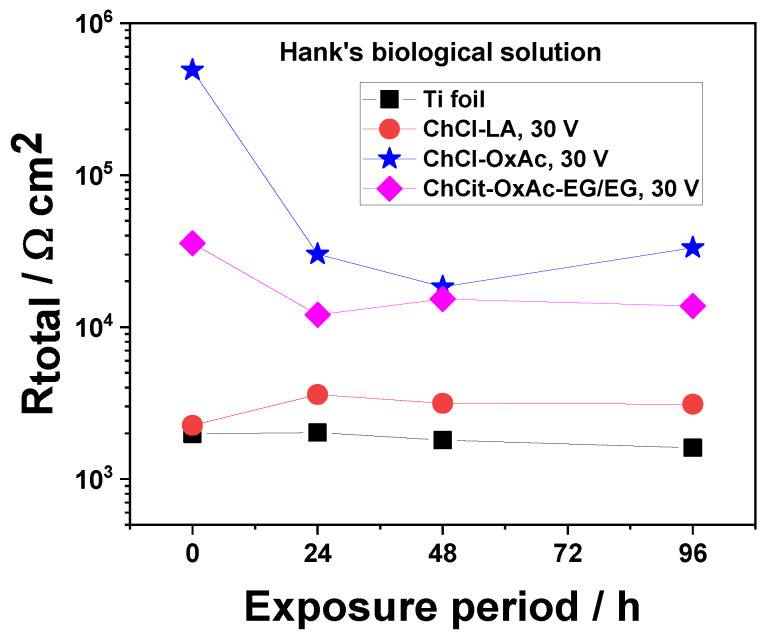
Evolution of Rtotal (Rtotal = R_ox_ + R_ct_) for anodic TiO_2_ thin layers obtained from DES systems during continuous immersion test in Hank’s biological solution.

Figure 14 illustrates the dependence of the R_total_ against the immersion period in Hank’s solution. It can be clearly seen that the anodic oxide layers prepared in ChCl-OxAc and ChCit-OxAc-EG/EG electrolytes provide the best corrosion protection, materialized by about one order of magnitude higher than those involving ChCl-LA electrolytes, even after 96 h of continuous immersion.

All of the results detailed above suggest that the preparation of colored anodic titanium oxide thin layers using various DES-based formulations at room temperature can provide an adequate corrosion performance for the metallic substrate, in addition to a wide color palette.

## 4. Conclusions

Following the findings obtained in this study, several procedures have been proposed to produce colored anodized layers onto Ti metallic substrates using various deep eutectic solvent (DES)-based formulations. Furthermore, new electrolyte systems based on choline chloride–oxalic acid and choline dihydrogen citrate–oxalic acid–ethylene glycol mixtures have been developed.

The applied voltage and the DES-based electrolyte formulation influence the formation of thin anodic layers showing different morphologies.

The achieved chromatic scale consists of the following order of colors: gold—blue—light blue—light blue/green—pink—violet. This depends on the applied potential and on the DES-based electrolyte. The films exhibit a relatively high level of brightness and color saturation. The hue vs. anodization voltage diagrams suggest that the oxide growth shows an almost linear dependence on the applied voltage.

The corrosion performance of the prepared anodic titanium oxide layers was assessed through polarization measurements and EIS for long immersion periods of 240 h in 0.5 M NaCl and of 96 h in Hank’s solution. The anodic titanium oxide layers produced in ChCl-OxAc and ChCit-OxAc-EG/EG electrolytes at 30 V for 3 min showed the best corrosion performance. Corrosion currents of around 1–2 µA cm^−2^ and total resistances of about 7 MΩ cm^2^ have been determined after 240 h of continuous immersion in 0.5 M NaCl, with no surface and color modifications.

Nevertheless, additional investigations are planned to enable a more detailed characterization of the colored anodic layers and to deepen the understanding of the layer-formation mechanisms associated with the observed morphology. Moreover, studies regarding color reproducibility are to be carried out as well.

## Figures and Tables

**Table 1:** Ionic liquid systems used for Ti anodic coloring.

System Abbreviation	Electrolyte Composition
ChCl-LA	ChCl: LA 1:2 (molar ratio)
ChCl-OxAc	ChCl: OxAc 1:1 (molar ratio)
ChCit-OxAc-EG	ChCit: OxAc: EG 1:1:1 (molar ratio)
ChCit-OxAc-EG/EG	ChCit-OxAc-EG/EG 1:1 (volumic ratio)

**Table 2:** Composition of biological Hank’s solution [29].

Chemical Compound	Concentration g/L
NaCl	8.8
KCl	0.4
CaCl_2_·2H_2_O	0.35
Na_2_HPO_4_·H_2_O	0.25
MgCl_2_	0.19
MgSO_4_·7H_2_O	0.06
C_6_H_12_O_6_	1

**Table 3:** The chemical composition of the colored titanium oxide layers obtained by anodic oxidation in the investigated DES-based electrolytes.

Electrolyte Type	Anodization Voltage, V	EDX Content, wt.%		
		Ti	O	C
ChCl-LA	10	84.96	12.38	2.66
	30	76.50	21.97	1.53
	60	69.82	27.56	2.62
ChCl-OxA	10	86.16	12.61	1.23
	30	70.83	26.15	3.02
	60	70.93	27.50	1.57
ChCit-OxAc-EG/EG	10	79.23	18.42	2.35
	30	66.51	31.07	2.43
	60	65.04	33.75	1.21

**Table 4:** Oxide growth rates obtained by linear interpolation of experimental data.

Electrolyte Type	Surface Finishing	Oxide Growth Rate nm/V	R^2 a^
ChCl-OxAc	Pickling	2.1	0.996
ChCl-LA	Pickling	1.9	0.994
ChCit-OxAc-EG/EG	Pickling	1.6	0.991

^a^ linear regression coefficient.

**Table 5:** Characteristic values resulting from polarization curve experiments in 0.5 M NaCl.

Coating System	Initial		240 h	
	E_cor_, V vs. Ag/AgCl	i_cor_, μA/cm^2^	E_cor_, V vs. Ag/AgCl	i_cor_, μA/cm^2^
Ti foil	−0.356	9.03	−0.61	15.7
ChCl-LA, 30 V	−0.552	5.32	−0.56	7.03
ChCl-OxAc, 30 V	−0.460	2.15	−0.589	2.45
ChCit-OxAc-EG/EG, 30 V	−0.49	6.01	−0.505	0.866

**Table 6:** Fitting results for impedance spectra for anodic TiO_2_ thin layers obtained from DES systems after exposure to 0.5 M NaCl solution for different times using the equivalent circuits proposed in Figure 10.

Conditioning Period, h	ChCl-LA		ChCl-OxAc		ChCit-OxAc-EG/EG	
	R_ox_, Ω cm^2^	R_ct_, Ω cm^2^	R_ox_, Ω cm^2^	R_ct_, Ω cm^2^	R_ox_, Ω cm^2^	R_ct_, Ω cm^2^
0	102	8324	437,708	-	16,841	580,000
24	35	1117	367	18,398	9379	15,264
48	25	7413	729	14,021	22	11,865
96	1490	14,086	7.39 × 10^6^	-	12,883	41,636
168	3513	214,100	7.76 × 10^7^	-	206	96,636
240	1337	29,690	149	41,915	861	7.81 × 10^6^

**Table 7:** Characteristic values resulting from polarization curve experiments in Hank’s biological solution.

Coating System	Initial		96 h	
	E_cor_, V vs. Ag/AgCl	i_cor_, μA/cm^2^	E_cor_, V vs. Ag/AgCl	i_cor_, μA/cm^2^
Ti foil	−0.452	20.4	−0.578	18.0
ChCl-LA, 30 V	−0.704	17.4	−0.713	10.0
ChCl-OxAc, 30 V	−0.662	0.99	−0.671	1.38
ChCit-OxAc-EG/EG, 30 V	−0.620	2.38	−0.612	2.54

## Data Availability

The original contributions presented in this study are included in the article/Supplementary Material. Further inquiries can be directed to the corresponding author.

## Supplementary Materials

The following supporting information can be downloaded at https://www.mdpi.com/article/10.3390/ma19061087/s1, Figure S1: Reflectance spectra of interference colors obtained during Ti anodization in ChCl-LA electrolyte by increasing the voltage from 10 V to 60 V. Figure S2: Reflectance spectra of interference colors obtained during Ti anodization in ChCit-OxAc-EG/EG electrolyte by increasing the voltage from 10 V to 80 V. Figure S3: The color palette obtained by anodic oxidation in the investigated DES-based electrolytes on Ti foils and screws.
