# Supplementary material for: Colored Anodic Titania Thin Layers Involving Various Deep Eutectic Solvent Formulations—Evaluation of Corrosion Behavior
Source: Materials (Basel). 2026 Mar 12;19(6):1087. doi: 10.3390/ma19061087 (PMC13027551; doi:10.3390/ma19061087)
Supplement: Supplementary file 1 [file materials-19-01087-s001.zip › materials-4156659-supplementary.pdf]

Article

Colored Anodic Titania Thin Layers Involving Various Deep Eutectic Solvent Formulations – Evaluation of Corrosion Behavior

Sabrina State (Rosoiu) [1,2], Adrian-Cristian Manea [3,4], Oana Brincoveanu [2], Veronica Anastasoaie [2], Liana Anicai [4] *

1 Faculty of Medical Engineering, National University of Science and Technology Politehnica Bucharest, 132 Calea Grivitei, 010737 Bucharest, Romania; sabrina.rosoiu@upb.ro

2 National Institute of Research and Development in Microtechnologies-IMT, 126A Erou Iancu Nicolae Str., 077190 Voluntari-Ilfov, Romania; oana.brincoveanu@imt.ro (O.B.); veronica.anastasoaie@imt.ro (V.A.)

3 Department of Inorganic Chemistry, Physical Chemistryand Electrochemistry, National University of Science and Technology Politehnica Bucharest, 132 Calea Grivitei, 010737 Bucharest, Romania; adrianmanea@catedra.chfiz.pub.ro

4 Center of Surface Science and Nanotechnology, National University of Science and Technology Politehnica Bucharest, Splaiul Independentei 313, 060042 Bucharest, Romania

* Correspondence: liana.anicai@cssnt-upb.ro

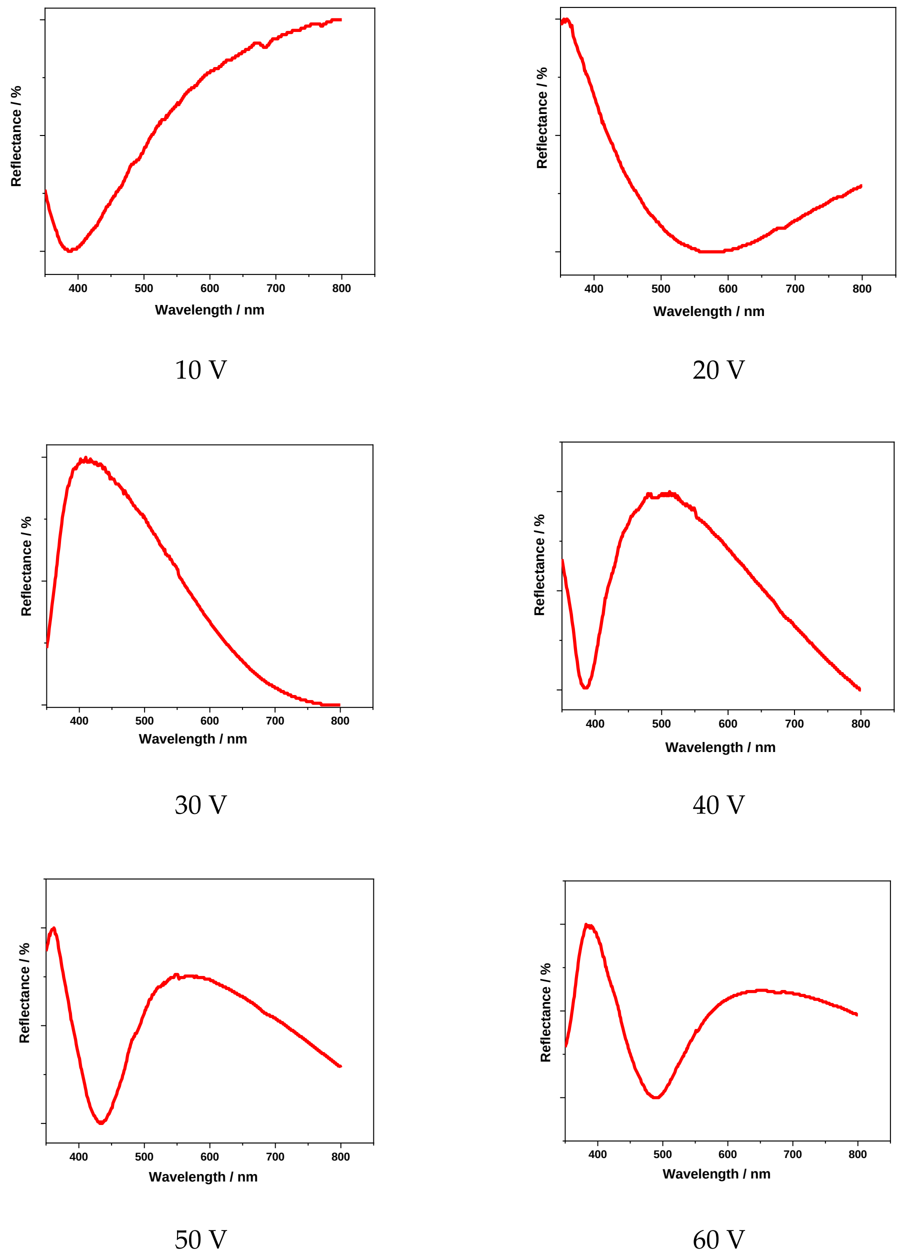

Figure S1. Reflectance spectra of interference colors obtained during Ti anodization in ChCl-LA electrolyte by increasing the voltage from 10 to 60 V

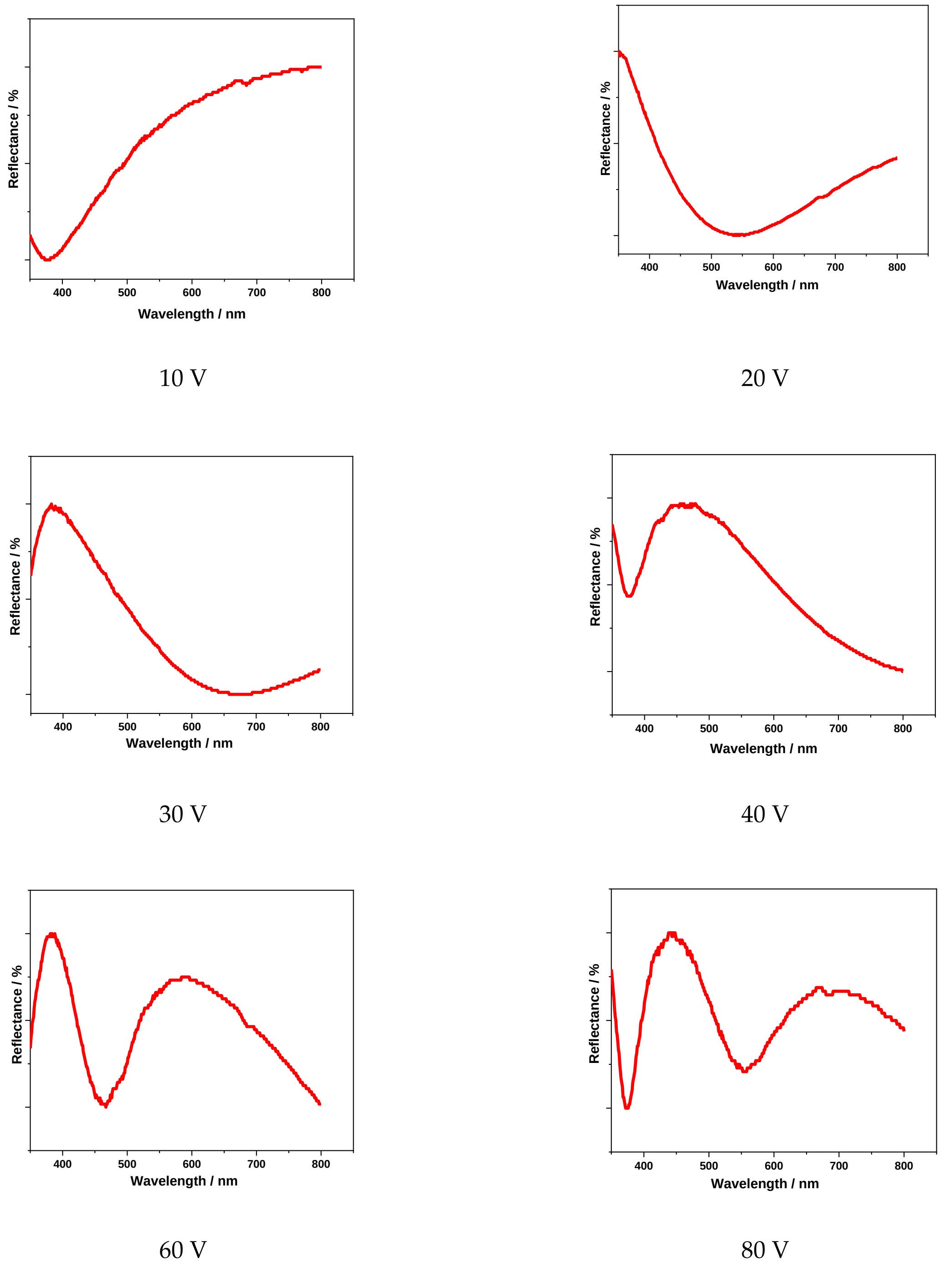

Figure S2. Reflectance spectra of interference colors obtained during Ti anodization in ChCit-OxAc-EG/EG electrolyte by increasing the voltage from 10 to 80 V

ChCl-LA, Ti counterelectrode, mild stirring, 25-27 ^{o}C, 3 min.

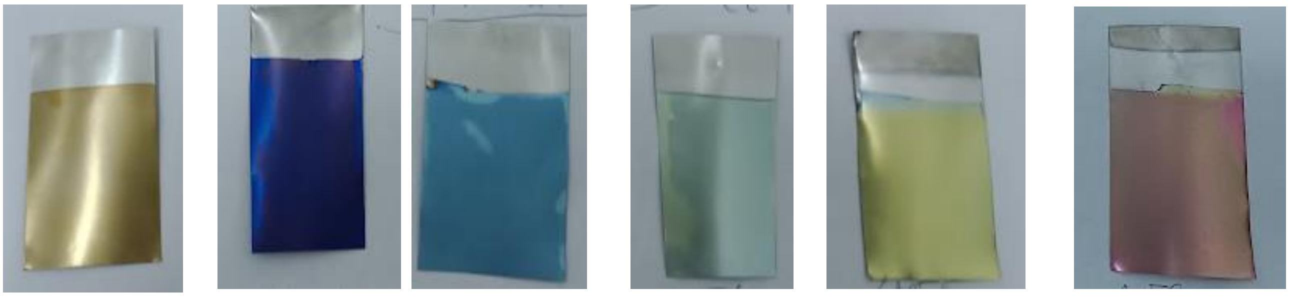

10 V | 20 V | 30 V | 40 V | 50 V | 60 V

ChCl-OxAc, Ti counterelectrode, mild stirring, 25-27 ^{o}C, 3 min.

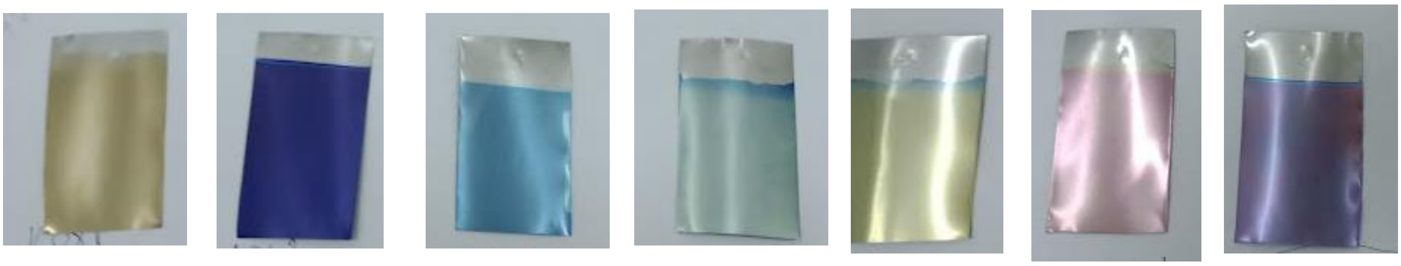

10 V | 20 V | 30 V | 40 V | 50 V | 60 V | 70 V

ChCit-OxAc-EG/EG, Ti counterelectrode, mild stirring, 25-27 ^{o}C, 3 min.

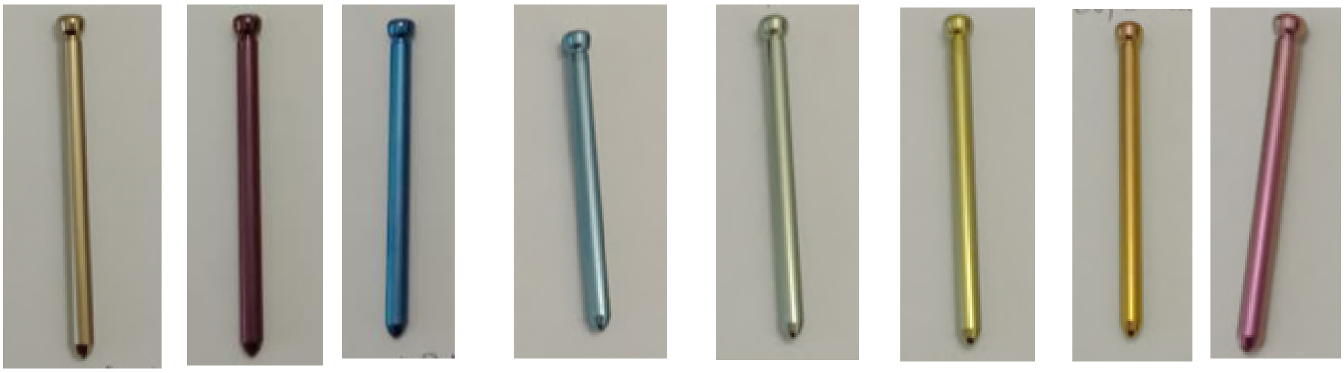

10 V | 20 V | 30 V | 40 V | 50 V | 60 V | 70 V | 80 V

Figure S3. The color palette obtained by anodic oxidation in the investigated DES-based electrolytes on Ti foils and screws
